# Proliferative Tumor Doubling Times of Prostatic Carcinoma

**DOI:** 10.1155/2011/301850

**Published:** 2011-09-21

**Authors:** Priya N. Werahera, L. Michael Glode, Francisco G. La Rosa, M. Scott Lucia, E. David Crawford, Kenneth Easterday, Holly T. Sullivan, Rameshwar S. Sidhu, Elizabeth Genova, Tammy Hedlund

**Affiliations:** ^1^Department of Pathology, University of Colorado Anschutz Medical Campus, P.O. Box 6511, Aurora, CO 80045, USA; ^2^Medical Oncology, University of Colorado Anschutz Medical Campus, CO 80045, USA; ^3^Radiation Oncology, University of Colorado Anschutz Medical Campus, CO 80045, USA; ^4^Pharmacy Department, University of Colorado Anschutz Medical Campus, CO 80045, USA

## Abstract

Prostate cancer (PCa) has a variable biology ranging from latent cancer to extremely aggressive tumors. Proliferative activities of cancers may indicate their biological potential. A flow cytometric assay to calculate maximum proliferative doubling times (*T*_max_) of PCa in radical prostatectomy specimens after preoperative *in vivo* bromodeoxyuridine (BrdU) infusion is presented. Only 4/17 specimens had tumors large enough for flow cytometric analysis. The *T*_max_ of tumors was similar and ranged from 0.6 to 3.6 months. Tumors had calculated doubling times 2- to 25-fold faster than their matched normal tissue. Variations in labeling index and *T*_max_ were observed within a tumor as well as between different Gleason grades. The observed PSA doubling times (PSA-DT) ranged from 18.4 to 32.0 months, considerably slower than the corresponding *T*_max_ of tumors involved. While lack of data for apoptotic rates is a limitation, apparent biological differences between latent versus aggressive PCa may be attributable to variations in apoptotic rates of these tumors rather than their cell proliferative rates.

## 1. Introduction

In the year 2010, an estimated 217,000 men were expected to be diagnosed with prostate cancer and 32,000 men to die from this disease in the United States alone [1]. Prostatic carcinoma (PCa) is a multifocal disease characterized by marked heterogeneity of morphology as well as clinical behavior. Nearly two-thirds of prostates contain multiple cancer foci and the index or the largest tumor may not necessarily determine the clinical outcome [2]. Autopsy studies show that >50% of men age 50 and older have histologic evidence of PCa [3]. These latent tumors are histologically identical to aggressive PCa, but have not progressed or became clinically evident in spite of their histological similarities. Aggressive PCa may be differentiated from latent carcinoma based on volume, histologic grade, and tumor doubling times [4, 5]. 

Histologic architectural grading is considered the main prognostic tool for PCa [6]. However, the rate of proliferation and tumor doubling time are also expected to have prognostic relevance, and is key to understanding the biological behavior of the tumor [5, 7, 8]. Research in this direction has been limited due to difficulties of obtaining accurate cell kinetic data in clinical settings. Accurate prediction of tumor progression and patient survival is a challenging problem in the clinical management of prostate cancer.

Knowledge of the biological behavior of latent and aggressive tumors will assist clinicians in customizing the treatment modalities. The current concept is that two major categories of prostatic carcinomas exist: (1) those that are latent and will not become clinically significant in a patient's lifetime, and (2) clinically significant tumors that invade and have the potential to metastasize to distant sites causing death. Prostate tumors of widely varying volumes (range 0.001–35 cc) can be found in a single prostatectomy specimen [2]. It is often assumed that the “small” volume tumors represent the slow growing latent ones while the “large” volume tumors represent the fast growing clinically significant ones. There is no absolute volume that defines “small” from “large” tumors. In addition, (a) small volume tumors can be of high grade and (b) even low-grade, low-volume carcinomas can be locally invasive [2]. While these categories imply that such tumors would have remarkably different doubling times, no data exist that directly measure this variable in appropriate tumors. 

The method commonly used to measure proliferative activity is evaluation of the S-phase fraction of the tumor [9]. This is carried out with autoradiography, which has certain limitations. Several investigators have used bromodeoxyuridine (BrdU) incorporation in ethanol fixed prostate biopsy tissue for the study of S-phase fraction or potential doubling times of PCa [10, 11]. Potential doubling times of 23–61 days were observed, but low labeling indices of PCa were a confounding problem [11]. The present study was undertaken to establish a technique to isolate nuclei from formalin-fixed paraffin-embedded radical retropubic prostatectomy (RRP) specimens from patients who were infused with BrdU prior to surgery. These specimens were used to calculate maximum proliferative doubling times (*T*_max_) of PCa by flow cytometric analysis.

## 2. Materials and Methods

This study was approved by the Colorado Multiple Institutional Review Board at the University of Colorado, Denver. Between August 1999 and April 2002, written informed consent was obtained from 17 RRP patients. The BrdU solution of 25 mg/mL was diluted into 250 mL of normal saline. The dose strength was 200 mg per m^2^ of body surface area. Between 20 and 48 hours prior to surgery, patients received BrdU by IV administration over a 30 min period. The starting and ending times of infusion were recorded, as well as the time of surgical removal of the prostate. Excised prostates were fixed in formalin, serially sectioned into 4 mm thick blocks, and paraffin-embedded for whole-mount section preparation [12]. From the proximal surface of each paraffin block, two 5-micron sections were cut. One set of 5-micron sections from each block was stained with hematoxylin and eosin (H&E) for routine histologic examination by a pathologist. The remaining set of 5-micron sections was used for immunohistochemical staining of BrdU to confirm nuclear incorporation. The boundary of the Gleason grade of each tumor focus was outlined in ink on H&E slides and transferred to acetate maps to generate 3D computer models of prostates as previously described [13, 14]. Biomorphometric data including multifocality, tumor volume, Gleason composition, capsule perforation were extracted from the 3D computer models of each RRP specimen [14]. Next, 3 sets of alternating 50-micron and 5-micron sections were cut from the proximal surface of each paraffin block containing the tumors of interest for doubling time analyses. Each 5-micron section was again H&E stained and mapped as described above. This was necessary to confirm the presence of tumors as they progressed through the blocks, and to track shifting positions of these tumors. Each 50-micron section was laid on top of the H&E slides containing the outlined tumors. After tracing the edges of the tumors onto the thick sections, tumors were excised using a razor blade. The Gleason score of all tumors were noted. Areas of relatively pure smooth muscle were also marked and excised for use as normal diploid, slow-growing controls for each patient's prostate. We define proliferative tumor doubling time *T*_max_ as the theoretical maximum doubling time assuming no cell death. The excised tissues were processed as follows.

### 2.1. Deparaffinization and Rehydration

Tissue samples were placed in Eppendorf centrifuge tubes and paraffin was removed by incubating for 3 min with Americlear (Richard-Allan Scientific, Kalamazoo, MI). Samples were centrifuged for 2 min at 389 ×g to gently pellet the tissue, and Americlear was removed. The washes with Americlear were repeated twice more. Residual Americlear was removed from the tissue with 2 changes of 100% ethanol, vortexing gently, and incubating 3 min each prior to centrifugation. Tissue was resuspended in fresh 100% ethanol (0.5 mL), and distilled water was added dropwise, tapping the tube between drops, to slowly rehydrate to a final volume of 1 mL (note: if tissue contains residual Americlear, the supernatant becomes cloudy upon the addition of water, and requires additional ethanol washes before attempting rehydration). After centrifugation, tissue was washed once in distilled water, centrifuged again, and supernatant removed.

### 2.2. Pepsin Digestion

Samples were resuspended in 1 mL 0.5% pepsin in 0.9% NaCl (pH 1.5), and incubated for 30 min at 37°C, vortexing after 15 min. After centrifugation to pellet cells, the supernatant was removed and fresh pepsin added. The suspension was pipetted to break up clumps of cells, and then incubated for 30 min at 37°C. After vortexing, the samples were allowed to continue to digest overnight at 4°C. Cells were then centrifuged and washed once with phosphate buffered saline (PBS) with pH 7.4 and once with distilled water, leaving approximately 150 *μ*L of supernatant on each pellet prior to tapping to resuspend.

### 2.3. Acid Denaturation of DNA

While slowly vortexing each sample, 1 mL of 2 M HCl with 0.5% triton X-100 was added dropwise. If added too quickly, nuclei may lyse. Samples were then incubated at room temperature for 30 min, centrifuged at 389 ×g for 5 min, and neutralized with 1 mL 0.1 M sodium borate, pH 8.5. PBS (1 mL) was added to each tube, and the suspension was run through 35 *μ*M cell strainers to remove undigested debris. Cells were then centrifuged and washed once in PBS with 0.1% bovine serum albumin and 0.5% Tween-20 (PBS-AT).

### 2.4. Antibody Staining

After removing most of the supernatant from each sample, the volume in each tube was adjusted to exactly 182.5 *μ*L with PBS-AT, and 87.5 *μ*L of each were transferred to new tubes for paired nonspecific controls. For BrdU detection, 5 *μ*L mouse monoclonal anti-BrdU antibody (clone PrB1, FITC-labeled, Phoenix Flow Systems Inc., San Diego, CA) was added to one set of tubes, yielding a final antibody concentration of 12.5 *μ*g/mL in 100 *μ*L. To measure nonspecific antibody staining, the remaining tubes received 12.5 *μ*L FITC-labeled mouse IgG1 (clone Dak-G01, Dako Corp., Carpenteria, CA) to yield 12.5 *μ*g/mL in 100 *μ*L. After mixing gently, tubes were incubated for either 40 min at room temperature in the dark (mixing after 20 min), or overnight at 4°C in the dark (mixing after 1 hour). Samples were washed 3 times in PBS-AT, incubating for 20–30 min at room temperature each time before centrifuging. These incubations are critical to allow diffusion of unbound antibody from the denatured DNA. The final cell pellet was resuspended in 0.4 mL PBS with 10 *μ*g/mL propidium iodide and 0.05 mg/mL DNase-free RNase A. Samples were then incubated for 1–3 hours at 4°C (protected from light) to allow intercalation of propidium iodide and degradation of any remaining RNA.

### 2.5. Flow Cytometric Analyses

Samples were analyzed for red and green fluorescence as first reported by Begg et al. [9] on a Coulter XL flow cytometer (Coulter-Beckman, Fullerton, CA). Listmode data were obtained using 50,000 cells for most samples, and at least 10,000 cells for small tumor specimens. All initial listmode files were more carefully analyzed subsequently using Cytomation Summit software (Cytomation Inc., Fort Collins, CO) to fine-tune all gates and statistical regions. Three independent analyses of the same listmode data were done for each sample, to account for differences in how gates and statistical regions were drawn. The following values were collected from the flow cytometric histograms.

**Figure 1:**
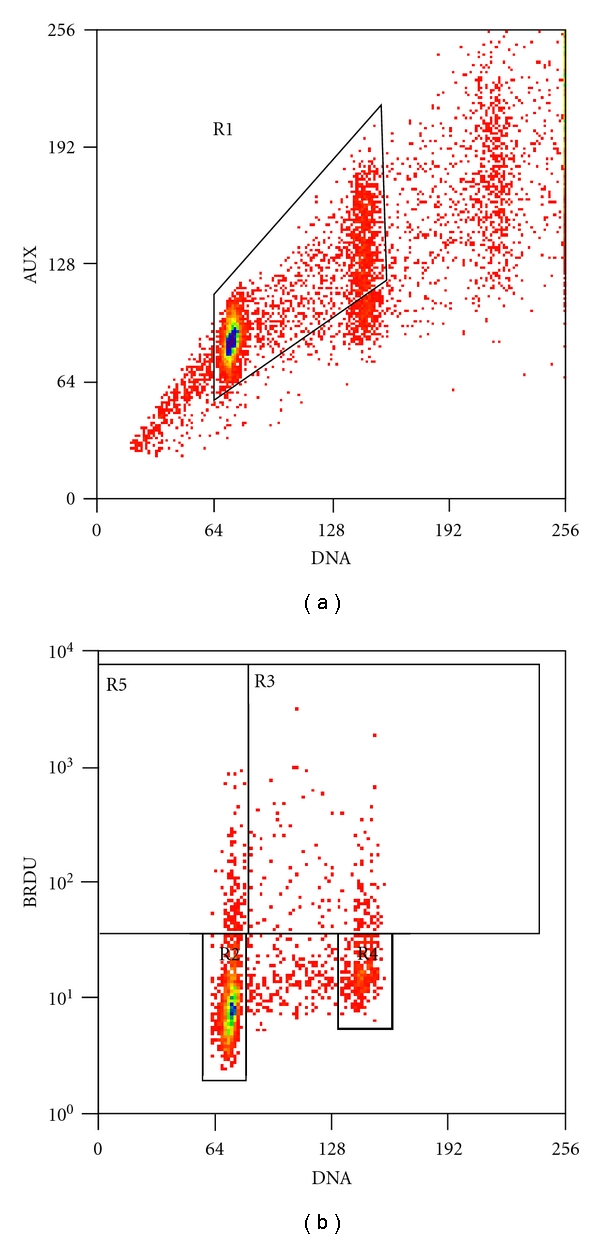
(a) Histogram 1: auxiliary peak red fluorescence versus red fluorescence, and (b) histogram 2: BrdU-FITC green fluorescence versus propidium iodide red fluorescence.

**Histogram 1 (Figure 1(a))**

Doublet discrimination was attempted by gating tightly on the G0/G1, S-phase, and G2/M populations. This was likely to exclude hypertetraploid cells if they existed, as well as doublets.

**Histogram 2 (Figure 1(b))**

Four different measurements were obtained from this histogram: FG1, FG2/M, FL, and LI. FG1 estimates the degree of red fluorescence (measured by mode rather than mean) of the BrdU-negative G0/G1 population. BrdU-positive cells are excluded from this measurement. FG2/M measures the red fluorescence (mode) of the BrdU-negative G2/M population. FL measures the mean red fluorescence of the BrdU-positive cells in S-phase and G2/M (exclude G0/G1). LI is the labeling index, measuring the percentage of all BrdU-positive cells.

The potential doubling times for each sample were then calculated using Begg's et al. original formulas [9], the flow cytometric values, and the length of time between the BrdU infusion and the surgical removal of the prostate (*T*_*c*_);

(1) $$\begin{array}{cc} & \text{Relative movement:}\;\\ & \;\;\text{RM}=\;\frac{\left[\text{FL}-\text{FG}1\right]}{\left[\text{FG}2/\text{M}-\text{FG}1\right]},\\ & \text{Length of S-phase:}\\ & \;\;\;T_{s}\;=\frac{\left[0.5\left(T_{c}\right)\right]}{\left[\text{RM}-0.5\right]},\\ & \text{Maximum proliferative doubling time:}\\ & \;\;T_{\max\;}\;=\frac{\lambda \left(T_{s}\right)}{\text{LI}}\;\;\;\text{where}\;\lambda \approx 1. \end{array}$$

### 2.6. Immunohistochemical Staining of BrdU and Ki-67

Two sets of consecutive 5-micron sections from paraffin-embedded blocks of RRP specimens were used in immunohistochemical (IHC) staining for BrdU and Ki-67 [15]. First set of 5-micron sections was baked in a 60°C oven for 1 hour. Rats infused with BrdU were sacrificed and the intestine harvested to serve as the tissue controls. After deparaffinization, antigen retrieval was performed in BORG solution, pH 9.5 (Biocare Medical, Concord, CA) for 5 min in the Decloaking chamber pressure cooker (Biocare). Slides were left on the countertop for 5–10 min to cool down at room temperature. Endogenous peroxidase was blocked with aqueous 3% hydrogen peroxide for 10 min. Slides were rinsed in APK wash (1X solution, Ventana Medical Systems, Tucson, AZ). All reactions were performed at room temperature. Test slides and positive control were incubated in anti-BrdU, 1 : 10, (*in-situ* kit, BD Biosciences, San Diego, CA) for 1 hour in a humidified chamber. A negative control was incubated with mouse ascites, 1 : 500, (Sigma Aldrich, St. Louis, MO). Slides were rinsed 3 times in APK for 5 min each time, and then further incubated in Streptavidin-HRP supplied from the *in-situ* kit for 30 min in a humidified chamber. A mixture of 1 mL DAB (diaminobenzidine) buffer and 1 drop DAB chromogen was incubated on the slides for 5 min. Afterwards, DAB buffer was rinsed off with deionized water. Slides were equilibrated in aqueous 1% acetic acid, stained in 0.02% light green SF yellowish for 5 dips, and returned to the acetic acid bath to set the color. Finally, slides were dehydrated in graded alcohols, cleared with xylene, and mounted with synthetic resin. 

IHC staining for Ki-67 was performed on the second set of 5-micron sections. A mouse antihuman antibody against Ki-67 (DAKO, Carpinteria, CA M7240; 1 : 300) was used to measure proliferation in the tissue sections. Antigen retrieval in BORG solution, pH 9.5 (Biocare Medical, Concord, CA, BDS1000G1) was performed for 5 minutes in the Decloaker pressure cooker (Biocare) at 125°C (22 psi). All incubations were accomplished by the Ventana NexES (Ventana Medical Systems, Tucson, AZ) immunostainer at 37°C. A Ventana I-VIEW DAB detection kit was used to detect the antigens through universal secondary antibodies, streptavidin-horseradish peroxidase enzyme, and DAB visualization. The sections were removed from the immunostainer and counterstained in light green sf yellowish (Sigma-Aldrich, St. Louis, MO, L1886-25G; 0.04% w/v) for 10 seconds, quickly dehydrated in graded alcohols, cleared in xylene, and mounted with synthetic resin. BrdU and Ki-67 LI using IHC staining were determined by counting number of positively stained cells per 1000 cells.

## 3. Results

**Figure 2:**
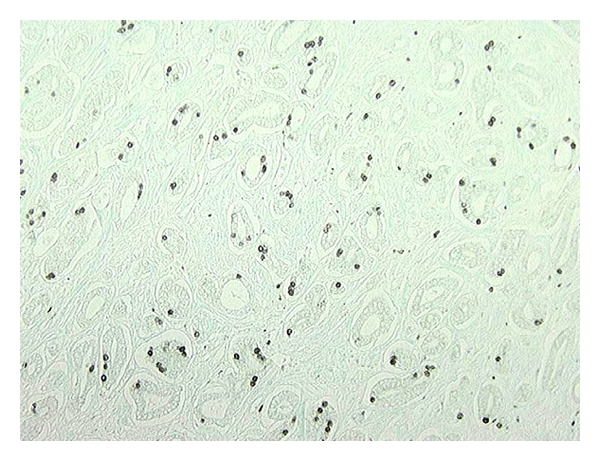
Gleason pattern 3 carcinoma with BrdU incorporated into the DNA of dividing cells (magnification 40X).

**Figure 3:**
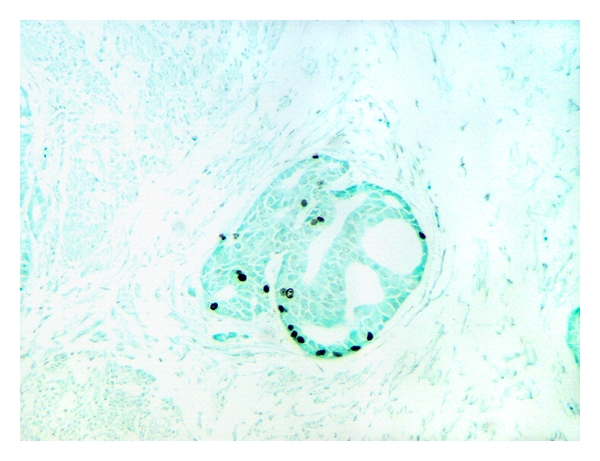
Distribution of BrdU incorporated cell nuclei within Gleason pattern 4 glands (magnification 100X).

The mean age of 17 patients was 57.6 ± 5.17 years (range 44–66 years) and the median age was 58 years. The mean prostate gland volume was 34.39 ± 10.45 cc (range 17.17–54.34 cc), the mean tumor volume was 1.62 ± 3.34 cc (range 0.001–8.663 cc), and the mean number of separate tumors was 2.6 (range 1–8). Only 4/17 prostates had tumors large enough for flow cytometric analysis. Tumor biomorphometry data of four prostates used in flow cytometric analyses are summarized in Table 1. The LI for BrdU and Ki-67 by IHC staining are given in Table 2. The mean LI for BrdU and Ki-67 was 2.14 ± 1.94% and 6.18 ± 4.27, respectively. There was no significant correlation between BrdU and Ki-67 LI (Pearson correlation coefficient *R* = 0.41, *P* = 0.16).  Figure 2 illustrates a photomicrograph of Gleason pattern 3 PCa where BrdU has been incorporated into the DNA of 5–7% of dividing S-phase cancer cells. A typical distribution of BrdU-incorporated cell nuclei within Gleason pattern 4 glands is illustrated in Figure 3.  Table 3 summarizes flow cytometry data for LI and *T*_max_ of prostate tumors from four prostates. The mean LI and *T*_*s*_ of prostate tumors and matched smooth muscle controls (in parenthesis) were 5.3 ± 3.1% (2.5 ± 0.28%) and 58 ± 27 hrs (224 ± 318 hrs), respectively. *T*_max_ of smooth muscle in different patients varied from 99 to 636 days. *T*_max_ for all tumors ranged from 19 to 108 days (0.6 to 3.6 months) and they doubled between 2-fold and 25-fold faster than their matched smooth muscle controls. 

The large tumor PBr4-T1 doubled approximately 2-3-times faster than the small tumor PBr4-T2 depending on the block from which the large tumor samples were excised. For example, T1 doubled approximately twice as fast as T2 when sampled from Block H but it doubled 3-times faster when sampled from Block F. Similar variations in the doubling rates were observed in the large tumor PBr18-T2. In this case, Gleason patterns 3 and 4 portions of T2 in Blocks B and D doubled twice as fast as the Gleason pattern 4 portion of the same tumor in Block D. However, the small tumor PBr18-T1 in Block B doubled 1.5–3-times faster than large tumor T2 in Blocks B and D. In the remaining two specimens (PBr6, PBr16), only the *T*_max_ of one large tumor each was reported since we did not have sufficient cell count to run flow cytometric analysis of corresponding small tumors. Also in PBr16-T1, analysis was limited to tumors excised from only two blocks (E and G). LI for smooth muscle in different specimens remained relatively constant but it varied among different tumors as well as within tumors. PSA Doubling times (PSA-DT) of these patients were also calculated [16] and are presented in Table 4 with corresponding average *T*_max_ for each tumor. Even in this small sample of tumors analyzed, *T*_max_ values of tumors involved were considerably faster than corresponding PSA-DT observed for each patient.

## 4. Discussion

We have developed an assay to use *in vivo* BrdU infused, formalin-fixed, paraffin-embedded RRP specimens for *T*_max_ evaluation by flow cytometric analysis. This is the first report to establish an *in vivo* BrdU incorporation technique in formalin-fixed prostate tumors and the calculation of *T*_max_ in patients with prostate cancer. This protocol appears to accurately estimate doubling times of tissues with rat intestine controls being the fastest (24–48 hr) and human prostate smooth muscle being the slowest. Our data demonstrate that it is possible to study proliferative activity of prostate tumors by direct measurement of *T*_max_. Our data also suggest there are variations in *T*_max_ calculations within a tumor depending on where the tumor was sampled. Similar variations in *T*_max_ were observed within a specific Gleason grade as well as among different Gleason grades. This variability observed in the proliferative activity of prostate tumors in regard to size and Gleason pattern may be due to (a) block-to-block variations in fixation, and (b) the heterogeneous nature of cancer cells and the multifocality of this particular disease. Since we used the average value of *T*_max_ calculated from three consecutive samples for each tumor section, contributions due to variability of the methodology should be small compared to other factors. Immunohistochemical staining confirmed that BrdU had been incorporated into the DNA of dividing PCa cells. Each tumor had 2% or more labeled cells, sufficient for flow cytometric analysis and *T*_max_ calculations. 

Out of 17 patients, we were able to analyze tumors of only four patients. The majority of the tumors were small (<0.1 cc) and hence we were unable to cut a sufficient number of 50-micron thick tissue sections for flow cytometric analysis. Several large tumors were used to develop and refine the flow cytometric protocol. This small sample size is one limitation of our study. 

Nemoto et al. studied the S-phase fraction of biopsies collected from patients with *in vivo* BrdU incorporation [10]. The biopsies were fixed with ethanol, embedded in paraffin, sectioned, and stained by an indirect immunoperoxidase method using anti-BrdU monoclonal antibody. LI was determined by counting the number of labeled cells. They demonstrated an average LI of Gleason grade 3, 2, and 1 PCa to be 4.37 ± 0.48%, 2.41 ± 0.49%, and 1.36 ± 0.39%, respectively. Haustermans et al. also used ethanol fixed biopsy tissue from patients with *in vivo* incorporation and reported potential doubling times from 23 to 61 days in prostate tumors among five patients [11]. 

In our study, the mean BrdU LI by IHC staining was 2.14 ± 1.94% and by flow cytometry 5.3 ± 3.1%. Ki-67 LI also measures cell proliferation. Nagao et al. found that prostate cancer patients with PSA > 4 had a mean Ki-67 LI of 10.5 ± 2.2% [15]. The mean Ki-67 LI in our study was 6.18 ± 4.27%. However, we did not find any significant correlation between BrdU and Ki-67 LI. Since *T*_max_ depends on both *T*_*s*_ and LI (given by the formula *T*_max_ = *λ*(*T*_*s*_)/LI), it is not possible to establish a direct correlation between *T*_max_ and LI alone. Consequently, LI of BrdU either by IHC or by flow cytometry alone are not accurate predictors of *T*_max_ and hence the biological potential of tumors. Our results suggest that the measurement of BrdU LI and *T*_max_ calculations by flow cytometry using formalin-fixed paraffin-embedded prostates may prove to be a quantitative assay of the biological potential of individual tumors. 

Schmid et al. found that PSA-DT were faster in patients with higher stages and grades [17, 18]. They found 20/28 clinically organ-confined cancers doubled at rates exceeding 4 years and concluded that prostate tumors have a constant (log-linear) growth rate that is very slow. It should be noted that in men with PSA levels between 4 and 10 with relatively small volume clinically localized tumors, the PSA levels do not correlate well with the tumor volume which potentially indicates that PSA levels in these patients may not be driven by the cancer cells themselves, but rather by other benign processes in the prostate such as inflammation and/or BPH [19]. In our study, total tumor volume of each prostate selected for flow cytometry was relatively large and hence PSA levels do correlate with tumor volume. PSA-DT for our patients were 18.4–32 months (552–960 days) [16]. This “apparent” slow growth rate of PCa tumors indirectly evaluated from PSA doublings may be attributed to concomitant cell death (apoptosis). Therefore, apoptosis is an important determinant of PSA-DT. Even though PCa cells are dividing at a faster rate, a relatively high apoptotic rate may result in a much slower net growth rate. However, there are no direct methods available to measure *in vivo* apoptotic rates of PCa as cells that undergo apoptosis are removed from the gland. Nevertheless, apparent biological differences between latent versus aggressive PCa may be attributable to variations in cell death rates of these tumors more than to their cell proliferative rates.

## 5. Conclusion

A flow cytometric assay using *in vivo* BrdU-infused, formalin-fixed paraffin-embedded RRP specimens was developed to determine *T*_max_ of prostate tumors. *T*_max_ of 4 PCa tended to be similar regardless of tumor volume or histologic grade. However, *T*_max_ of tumors were faster than observed PSA-DT of corresponding patients. While lack of data for apoptotic rates is a limitation of this study, relative variations in apoptotic rates may make the difference between latent and aggressive PCa, rather than *T*_max_. Future studies need to focus on tumor proliferative doubling times as well as apoptotic rates to better understand biological differences of latent versus aggressive prostate cancer.

## Figures and Tables

**Table 1:** Tumor biomorphometric data.

Specimen	Number of tumors	Gleason score	Tumor volume, cc	Volume of Gleason 3, cc	Volume of Gleason 4, cc
PBr4	1	3 + 3	2.823	2.823	
	2	3 + 3	0.121	0.121	
	3	3 + 3	0.022	0.022	
	4	3 + 3	0.006	0.006	
	5	3 + 3	0.015	0.015	
	6	3 + 3	0.001	0.001	

PBr6	1	3 + 3	0.656	0.656	
	2	3 + 3	0.009	0.009	
	3	3 + 4	0.037	0.025	0.012
	4	3 + 3	0.028	0.028	

PBr18	1	3 + 3	0.015	0.015	
	2	3 + 4	0.842	0.736	0.098
	3	3 + 3	0.755	0.755	
	4	3 + 4	0.023	0.009	0.014
	5	3 + 3	0.021	0.021	

PBr16	1	3 + 4	7.642	7.511	0.131
	2	3 + 3	0.022	0.022	

**Table 2:** BrdU and Ki-67 *LI* by IHC staining of consecutive 5-micron sections.

Specimen and block with tumor	*BrdU *LI % by IHC	*Ki67 *LI % by IHC
PBr1-E	3.3	4.6
PBr2-C	0.4	7.5
PBr3-D	1.2	0.9
PBr6-E	1.1	2.8
PBr7-E	3.5	8.9
PBr8-C	1.3	3.1
PBr9-G	0.1	9.5
PBr10-C	2.2	5.0
PBr12-E	5.0	7.9
PBr14-D	0.4	2.1
PBr15-C	0.7	1.9
PBr16-D	6.5	10.6
PBr19-C	2.1	15.6

**Table 3:** Proliferative tumor doubling times of prostatic carcinoma.

Specimen	Tumor	Tumor volume, cc	Block with tumor	Gleason pattern*	*BrdU *LI % by flow cytometry	*T*_max _, days	Growth rate**
PBr4	T1	2.823	T1-F	3	9.1 ± 0.9	25.7 ± 4.0	25X
			T1-G	3	4.2 ± 0.5	41.5 ± 3.9	15X
			T1-H	3	3.7 ± 0.3	48.0 ± 4.0	13X
	T2	0.121	T2-C	3	3.4 ± 0.3	97.9 ± 7.8	7X
	Muscle	—	—	—	2.6 ± 0.0	636.2 ± 135.1	—

PBr6	T1	0.656	T1-B	3	3.2 ± 0.3	50.7 ± 1.5	2X
			T1-C	3	3.1 ± 0.3	51.1 ± 5.7	2X
	Muscle	—	—	—	2.3 ± 0.4	98.6 ± 12.7	—

PBr18	T1	0.015	T1-B	3	5.7 ± 2.2	36.6 ± 7.3	6X
	T2	0.842	T2-B	3 & 4	5.2 ± 1.1	52.2 ± 7.0	4X
			T2-D	4	3.8 ± 0.9	108.1 ± 14.8	2X
			T2-D	3 & 4	3.7 ± 0.7	57.4 ± 8.9	4X
	T3	0.755	T3-D	3	13.6 ± 1.7	17.3 ± 2.6	13X
			T3-E	3	4.9 ± 0.7	73.5 ± 9.1	3X
	Muscle	—	—	—	2.7 ± 0.1	221.7 ± 41.8	—

PBr16	T1	7.642	T1-E	3	2.0 ± 0.1	60.0 ± 4.8	2X
			T1-G	4	7.8 ± 0.2	19.0 ± 1.7	7X
	Muscle	—	—	—	1.4 ± 0.2	137.0 ± 33.0	—

*Gleason pattern: Gleason pattern of the excised tumor section for a given block.

**Growth rate: *T*_max_ of matched muscle/*T*_max_ of tumor.

**Table 4:** Proliferative tumor doubling time versus PSA doubling time.

Specimen and Tumor number		Tumor volume (cc)	Average *T*_max _* (months)	PSA doubling Time (months)
PBr4	T1	2.823	1.28	18.4
	T2	0.121	3.26	

PBr6	T1	0.656	1.70	22.8

PBr18	T1	0.015	1.22	27.8
	T2	0.842	2.42	
	T3	0.755	1.51	

PBr16	T1	7.642	1.32	32.0

* is the average of *T*_max_ calculated when tumor is found in more than one paraffin block.
